# Negative Impact and Positive Value of Caregiving in Spouse Carers of Persons With Dementia in Sweden

**DOI:** 10.1093/geroni/igab046.586

**Published:** 2021-12-17

**Authors:** Marcus Johansson, Kevin J McKee, Lena Dahlberg, Martina Summer-Meranius, Christine Williams, Lena Hammar

**Affiliations:** 1 Dalarna University, Falun, Dalarnas Lan, Sweden; 2 Mälardalen University, Västerås, Vastmanlands Lan, Sweden; 3 Florida Atlantic University, Boca Raton, Florida, United States; 4 Mälardalen University / Dalarna University, Västerås, Vastmanlands Lan, Sweden

## Abstract

As welfare providers struggle to meet the care needs of persons with dementia (PwDs), most of their needs are being met by a family carers, most often a spouse. The situation for spouse carers is unique, e.g., with grief, loneliness and loss of intimacy combining with stress and poor health. Research is needed to develop adequate support for spouse carers based on evidence of what influences negative and positive outcomes of care. The present study investigated psychosocial correlates of spouse carers’ (i) negative impact and (ii) positive value of caring. Data from a cross-sectional survey of 165 spouse carers community-resident in Sweden was analysed in two hierarchical regression models to predict negative impact and positive value of caring. Results found that negative impact and positive value were explained by different variables, significant predictors for negative impact included carer stress, health, and emotional loneliness, and change in intimacy with the care-recipient, while positive value was predicted by mutuality, change in closeness to the care-recipient and quality of support. Negative impact and positive value shared variance of only 17.2%. Thus, negative impact and positive value represent different aspects of the carer situation. Consequently, support needs to target several aspects in carers’ life, aiming to; facilitate for spouses to manage PwD’s impairment, increase emotional support while also strengthening the relationship between carer and PwD to reduce negative impact while increasing positive value.

